# Renal replacement therapy-requiring acute kidney injury due to tubulointerstitial nephritis and uveitis syndrome: case report

**DOI:** 10.1186/s13256-021-03147-z

**Published:** 2021-12-20

**Authors:** B. Marahrens, K. Amann, K. Asmus, S. Erfurt, D. Patschan

**Affiliations:** 1 Zentrum für Innere Medizin 1, Universitätsklinikum Brandenburg, Medizinische Hochschule Brandenburg, Hochstraße 29, 14770 Brandenburg, Germany; 2 Division of Nephropathology, University of Erlangen, Erlangen, Germany

**Keywords:** AKI, RRT, Interstitial nephritis, TINU syndrome, Case report

## Abstract

**Background:**

Acute kidney injury is a major challenge for today’s healthcare systems around the globe. Renal replacement therapy has been shown to be beneficial in acute kidney injury, but treatment highly depends on the cause of the acute kidney injury. One less common cause is tubulointerstitial nephritis, which comes in different entities. A very rare type of tubulointerstitial nephritis is tubulointerstitial nephritis and uveitis syndrome, in which the patient presents with additional uveitis.

**Case presentation:**

A 19-year-old caucasian male presented with mild dyspnea, lack of appetite, weight loss, and moderate itchiness. Lab results showed an acute kidney injury with marked increase of serum creatinine. The patient was started on prednisolone immediately after admission. As the patient in this case showed symptoms of uremia on admission, we decided to establish renal replacement therapy, which is unusual in tubulointerstitial nephritis and uveitis syndrome. During his course of dialysis, the patient developed symptoms of sepsis probably due to a catheter-related infection requiring intensive care and antibiotic treatment, which had to be terminated early as the patient developed a rash. Intensified immunosuppression, combined with antibiotics, significantly resolved excretory kidney dysfunction.

**Conclusions:**

Since both the primary inflammatory process and the secondary infectious complication significantly impaired excretory kidney function, kidney function of younger individuals with new-onset anterior uveitis should be monitored over time and during follow-up.

## Introduction

Acute kidney injury (AKI) substantially worsens the prognosis of hospitalized patients worldwide. The in-hospital incidence increased over the last decades and varies between 5% and 20%, with an average mortality of 5–10% [1–3]. However, the mortality risk dramatically increases under intensive care conditions, reaching 50% or even more [4]. AKI has been identified as an independent risk factor for death at the intensive care unit (ICU) [5]. However, one must keep in mind that there are different definitions of AKI and the incidences vary depending on the criteria used [6]. Among the causes, renal hypoperfusion of various origin is the most frequent. Regarding less frequent etiologies, acute tubulointerstitial nephritis (ATIN) accounts for 2–27% of all AKI events in the hospital [7, 8]. ATIN is most likely underdiagnosed since it may exclusively be associated with urine abnormalities such as tubular proteinuria or isolated or combined electrolyte loss. In more severe cases, subjects potentially suffer from systemic manifestations also (fever, rush, myalgia/arthralgia) [9]. The most common cause of ATIN is transient or regular administration of a certain type of drug. Proton pump inhibitors, beta-lactam antibiotics, and allopurinol have been identified to be responsible with higher probability, although in theory, any particular drug can cause ATIN. Other causes are systemic disorders, electrolyte disturbances, and infections [10]. Tubulointerstitial nephritis with uveitis (TINU) syndrome is rarely responsible for ATIN. The disease was initially described in the mid-1970s [11]. Since then, hardly 300 cases have been reported worldwide.

Herein, we present the case of a 19-year-old male patient who developed renal replacement therapy (RRT)-requiring AKI due to TINU syndrome. The second part will summarize information about both the epidemiology and etiology of TINU syndrome. Additionally, data on RRT in TINU-associated AKI will be discussed.

## Case report

**Fig. 1:**
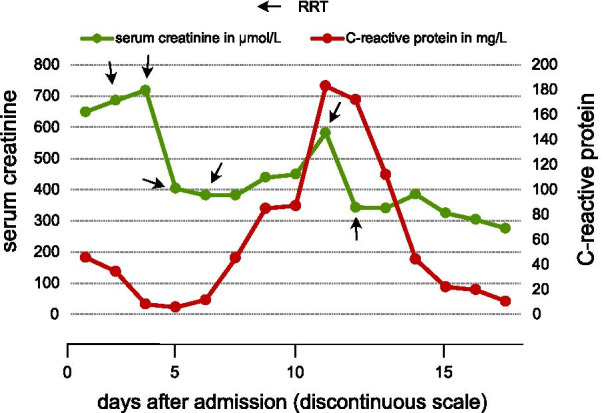
Lab results and creatinine/estimated glomerular filtration rate (eGFR). On admission, serum creatinine of our patient was 649 µmol/l and prednisolone treatment was started. Serum creatinine decreased markedly after the first two sessions of RRT. It increased again when the patient developed catheter-associated infection around day 10. After the last session of RRT on the 12th day after admission, serum creatinine levels kept falling in our patient and reached around 200 µmol/l when he was discharged

In May 2021, a 19-year-old caucasian male patient was referred to the university hospital of Brandenburg owing to a severe decline of excretory kidney function. The serum creatinine concentration was 649 µmol/l (normal range 62–106 µmol/l) at the time of admission (Fig. 1). The patient suffered from mild dyspnea, lack of appetite, and moderate itchiness.

The patient did not report any known diseases, nor did he take any medication on a regular basis. Approximately 10 weeks earlier, he had an upper respiratory tract infection that did not require antibiotics or other medications such as nonsteroidal anti-inflammatory drugs (NSAIDs). Since then, he did not recover completely but instead suffered from persistent fatigue. Also, he lost 8 kg of body weight until admission. He denied fever, nausea/vomiting, myalgia, arthralgia, skin abnormalities, photosensitivity, Raynaud symptoms, and hair loss. He did not report morning stiffness or lower back pain. Three weeks before admission, he noticed pain in his right eye, accompanied by redness and blurred vision. A prompt ophthalmological examination led to the diagnosis of anterior uveitis. The ocular inflammatory process was not treated in a systemic manner, particularly not with systemic steroids, antibiotics, or NSAIDs. He exclusively received steroid-containing eye-drops. One day before admission, he underwent outpatient control of several blood parameters to identify the etiology of ocular inflammation. Serum analysis showed severely deteriorated kidney function.

At the time of admission, he presented an overall reduced physical condition. His height was 180 cm and body weight 93 kg (BMI: 28.7). His initial blood pressure was 144/114 mmHg and heart rate 124 beats per minute. Respiratory rate was 14 breaths per minute, and peripheral oxygen saturation was 99%. He had no increased body temperature. Examination of heart, lungs, and abdomen did not reveal any pathological findings, the same applied for both the central and peripheral nervous system. Abdominal skin was moderately affected by striae distensae.

Besides impaired excretory kidney function, the patient showed moderately increased C-reactive protein (CRP) (45.7 mg/l; normal range < 5 mg/l) and elevated haptoglobin (2.6 g/l; normal range 0.3–2.0 g/l). Also, parathormone (PTH) was mildly elevated (72.8 pg/ml; normal range 15–65 pg/ml). Immune diagnostics revealed the following positive findings: anti-nuclear antibodies (ANA) titer (1:160; normal range < 1:160) and anti-La (56.6; normal range < 46). Both cytoplasmic and perinuclear Anti-Neutrophil Cytoplasmic Antibodies (c- and pANCA) were negative, anti-proteinase 3 was 2.3 U/mL (normal range < 10 U/mL). Light chain (LC) diagnostics showed increases of both, kappa- and lambda-LC (121 mg/l; normal range 3.3–19.4 mg/l, and 60.1 mg/l; normal range 5.71–26.3 mg/l), and the ratio differed from the normal range as well (2.01; normal range 0.26–1.65). Total serum immunoglobulin-G (IgG) was mildly elevated (22.1 g/L; normal range 5.49–15.8 g/L). *Chlamydia pneumoniae*-IgG (21 RE/ml; normal range < 16 RE/ml) was positive, as was serological testing for Epstein–Barr virus (EBV) [virus-capsid antigen (VCA) EBV-IgG-antikoerper (Ak) (enzyme-linked immunosorbent assay) 137 RE/mL (normal range < 16 RE/mL), Epstein-Barr Nuclear Antigen 1 (EBNA 1)-IgG-Ak 1.02 (normal range < 80)]. Differential blood cell count showed an eosinophil percentage of 3.6% (normal range 0.5–7%). Other non-aberrant findings were monocytes, platelet count, and serum and urine calcium.

Semiquantitative urine analysis showed a proteinuria of 0.25 g/l and few erythrocytes (25/µl; normal: negative). The daily proteinuria was determined to be 0.77 g (normal range < 0.15 g). Urinary eosinophils were negative.

Transthoracic echocardiography showed a mildly reduced left ventricular ejection fraction (50%; normal range > 60%). Diastolic function was impaired, although mild as well. The inferior part of the left ventricle was akinetic. Visually, the right ventricular function was slightly reduced. Computed tomography of thorax and abdomen revealed diffuse intraabdominal lymph node expansion. The initial ophthalmological investigation confirmed the diagnosis of unilateral anterior uveitis of the right eye. Specifically, the right conjunctiva showed perilimbical hyperemia, and the cornea was unaffected. The anterior chamber was not flattened and did not contain relevant cell numbers. Retinal investigation did not reveal any signs of inflammation.

**Fig. 2:**
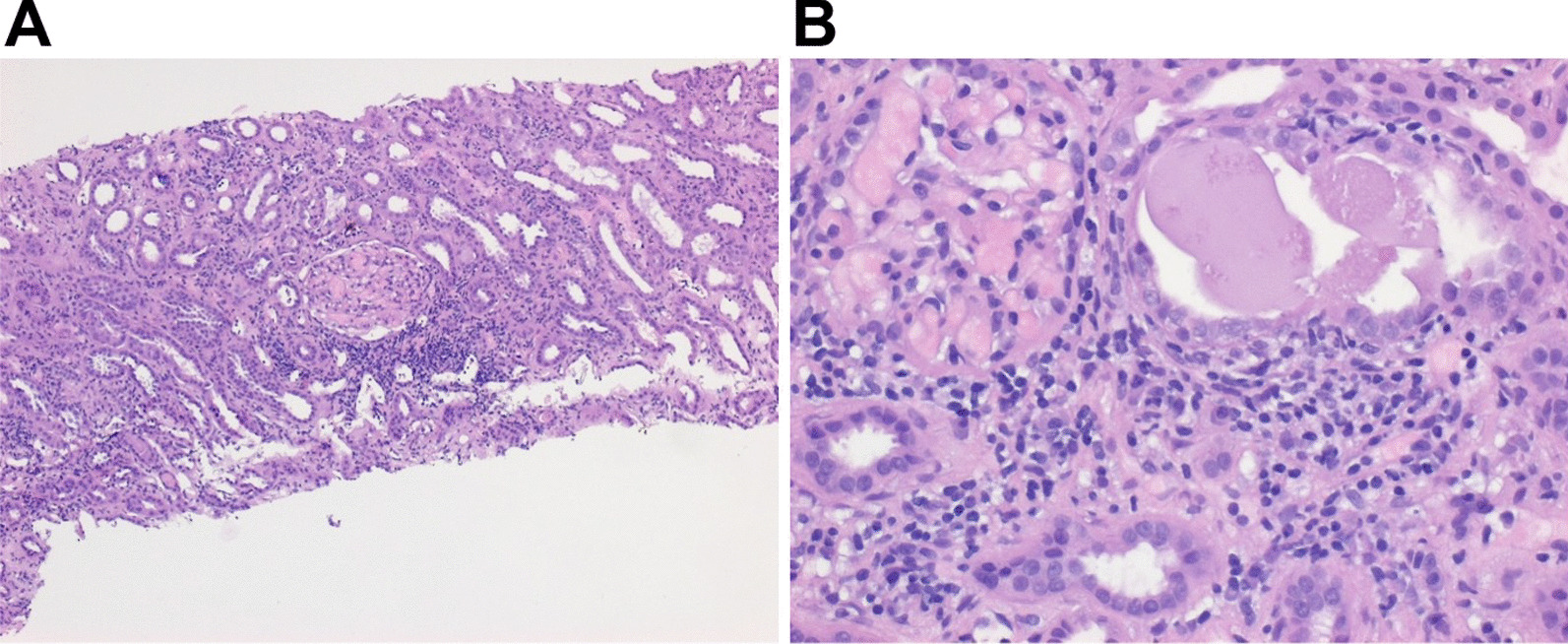
Kidney histology (hematoxylin–eosin staining). Both images (**A** and **B**) show the renal biopsy of our patient with TINU syndrome (magnifications: left panel 10×, right panel 40×), revealing interstitial inflammatory infiltrates around the tubuli mainly composed of lymphocytes

Due to AKI of unknown origin, we performed kidney biopsy (6 days after admission). Two samples were obtained from the left kidney. Initial ultrasound analysis showed normal organ dimensions and no signs of obstruction. The pathological investigation by an experienced renal pathologist showed interstitial inflammatory infiltrates around the tubuli mainly composed of lymphocytes (Fig. 2). The findings led to the diagnosis of acute interstitial nephritis [12]. Signs of glomerular inflammation were absent. The diagnosis was tubulointerstitial nephritis with anterior uveitis (TINU) syndrome of no specific or suspected origin.

Immediately after admission, the patient received intravenous glucocorticoids (prednisolone 250 mg daily) on three consecutive days, followed by oral prednisolone (1 mg/kg daily for 7 days, dose reduction of 10 mg daily every 7 days thereafter) since we initially suspected an ANCA-associated autoinflammatory disease. Also, we started the patient on renal replacement therapy (RR, hemodialysis) after central vein catheter insertion into the right femoral vein. Volume depletion during individual dialysis session was not mandatory since urine production was not affected. One week after admission, the patient suffered from fever and general weakness. He received intravenous antibiotics (piperacillin and tazobactam) and was transferred to the local intensive care unit (ICU). The central vein catheter was removed since the patient showed localized pain around the insertion area, that is, signs of catheter-related blood infection. RRT was continued after establishing a new central vein catheter at the ICU. Two days after the initial fever attack, the patient developed generalized rash including moderate itchiness. The antibiotics therapy was adapted to meropenem. During the ICU stay, oral prednisolone therapy was continued as initiated. Discharge from the ICU was initiated after 3 days. The last dialysis treatment session was performed 1 week before discharge from the hospital (Fig. 1). Kidney excretory function continuously improved, with a last serum creatinine concentration of 214 µmol/l. Also, the ocular manifestation resolved almost completely after local corticosteroid eye drop treatment for 7 days. The in-hospital stay lasted for nearly 3 weeks, and the further management was planned in the outpatient area.

## Discussion

Herein, we present the case of a 19-year-old male subject who suffered from dialysis-requiring AKI due to TINU syndrome, aggravated by catheter-associated systemic infection. The latter was promoted by immunosuppressive therapy for interstitial nephritis.

TINU syndrome was first described by Dobrin *et al*. [11]. They reported on two patients who presented with AKI (therein: renal failure) due to interstitial nephritis, accompanied by bilateral anterior uveitis. Also, subjects showed granuloma formation in the bone marrow and a substantial increase in humoral markers of inflammation. In 2001, Mandeville and colleagues [13] identified a total of 133 TINU patients described in the literature. Sixteen years later, Okafor *et al*. [14] reviewed epidemiological data of TINU syndrome in a detailed manner. They cited studies on uveitis etiologies in several countries and finally identified the syndrome to be prevalent in 0.1–2.0% of all subjects suffering from uveitis. On the other hand, ATIN can be found in 10% of all biopsies in AKI cases, and TINU syndrome accounts for 9–22% of ATIN cases [8, 15]. Meanwhile, several risk or etiological factors have been identified to cause the syndrome. For instance, the risk varies with age, with TINU syndrome being more prevalent in younger individuals. Both Mandeville [13] and Mackensen [16] reported an age median of 15 years. Okafor *et al*. [14] summarized several references on genetic factors potentially responsible for increasing the risk even further. Two etiological categories shall be discussed briefly: medications and infections. Proton pump inhibitors (PPI) have reliably been documented to increase the risk for interstitial nephritis in general [17]. Surprisingly, a literature search applying either the terms “TINU” and “PPI” or “TINU” and “proton pump inhibitors” reveals one particular reference: a review article on interstitial nephritis in rheumatic diseases. The missing association between PPI and TINU syndrome indicates a distinct pathogenesis of the syndrome, which apparently differs from the pathogenesis of incident ATIN without ocular manifestations. In contrast to PPI, nonsteroidal anti-inflammatory drugs (NSAIDs) have been associated with TINU syndrome by at least three authors [18–20]. However, whether the substances were responsible in a mechanistic manner is speculative in nature. In a larger case series study published by Li and colleagues in 2014 [21], the administration of the following antibiotics was reported prior to TINU syndrome manifestation: amoxicillin, cephalexin, cefoperazone, cefuroxime, meropenem, azithromycin, and levofloxacin. Overall, 19.4% of the patients had a positive drug history for at least one of the substances within 20 days before TINU onset. In very few subjects (3.2%), Chinese herbs were administered before disease onset. Among infections, viruses have been suggested to play the most critical roles in TINU induction [14]. At least two references indicate an association with Epstein–Barr virus infections [22, 23]. However, it always needs to be noted that serological abnormalities that potentially indicate (sub)acute virus infections also may occur as bystander phenomena. Our patient showed positive anti-EBV-IgG. His abdominal lymph node expansion might have occurred as late or persistent manifestation of infection with the virus weeks before onset. However, making a distinct decision is difficult since we did not perform a more detailed EBV analysis of the kidney specimen. It also needs to be mentioned that a drug-induced pathogenesis was very unlikely in our patient: he did not receive any systemic medications at all prior to admission.

TINU syndrome is not only a rare cause of tubulointerstitial nephritis, but in many cases renal function is reduced only marginally and the ophthalmologic symptoms are predominant. As in other types of ATIN, RRT in TINU syndrome is most likely not required as long as the diagnosis has been made in time. Regarding our patient, the disease onset arguably took place 10 weeks or even longer before admission to the hospital. A PubMed-based search for the terms “TINU” and “dialysis,” performed in July 2021, delivered only six references. One reference reported on 21 Japanese children with ATIN, three of who suffered from TINU syndrome [24]. In total, 4 out of 21 subjects required RRT because of AKI. In 2015, Hoste and colleagues [25] published a prospective investigation that analyzed numerous risk and outcome variables in subjects treated at a total number of 139 intensive care units in 33 countries. RRT became mandatory in 13.5% of the 1802 included patients. These data were acquired in critically ill subjects. Therefore, the RRT prevalence of all in-hospital AKI cases is supposedly lower. But even if it ranges around 10%, only very few AKI subjects require RRT because of ATIN. Assuming that 2–27% of all in-hospital AKI events are triggered/caused by ATIN [8, 9], and also assuming that ATIN results from TINU syndrome in 9–22% of cases [15], the percentage of all AKI subjects requiring RRT because of tubulointerstitial nephritis with anterior uveitis syndrome is marginal. It needs to be emphasized that every individual AKI episode increases the risk for chronic kidney disease (CKD) in the long term [26, 27]. Our patient required transient RRT, but the whole procedure, although undoubtedly life-saving, increased the CKD risk even further since he developed catheter-related blood infection. The latter was followed by an additional AKI episode as shown in Fig. 1. Therefore, he is at substantial CKD risk in the long term, although an exact risk prediction is still difficult to provide. We recommended controls of serum creatinine at least twice monthly after discharge. Finally needs to be mentioned that the diagnosis ATIN could have been suspected earlier since systemic prednisolone therapy was initiated immediately. Therefore, typical ATIN-associated findings such as eosinophil accumulation/appearance in blood/urine might have been disappeared. On the other hand, the severity of the disease did not allow the decision for or against steroids to be prolonged.

## Conclusion

In summary, we reported the case of a 19-year-old male patient with RRT-requiring AKI due to TINU syndrome of unknown etiology. The disease required intensified immunosuppressive therapy and transient dialysis. Since both the primary inflammatory process (ATIN) and the secondary infectious complication significantly impaired excretory kidney function, kidney function of younger individuals with new-onset anterior uveitis should be monitored over time and during follow-up.

## Data Availability

The data supporting the findings of this study are available from the corresponding author upon reasonable request at daniel.patschan@mhb-fontane.de.
